# Potential role and mechanism of IFN-gamma inducible protein-10 on receptor activator of nuclear factor kappa-B ligand (RANKL) expression in rheumatoid arthritis

**DOI:** 10.1186/ar3385

**Published:** 2011-06-27

**Authors:** Eun Young Lee, MiRan Seo, Yong-Sung Juhnn, Jeong Yeon Kim, Yoo Jin Hong, Yun Jong Lee, Eun Bong Lee, Yeong Wook Song

**Affiliations:** 1 Division of Rheumatology, Department of Internal Medicine, Seoul National University College of Medicine, 28 Yongon-Dong, Chongno-Gu, Seoul, 110-744, Republic of Korea; 2 Department of Biochemistry, Seoul National University College of Medicine, 28 Yongon-Dong, Chongno-Gu, Seoul, 110-744, Republic of Korea

## Abstract

**Introduction:**

IFN-gamma inducible protein-10 (CXCL10), a member of the CXC chemokine family, and its receptor CXCR3 contribute to the recruitment of T cells from the blood stream into the inflamed joints and have a crucial role in perpetuating inflammation in rheumatoid arthritis (RA) synovial joints. Recently we showed the role of CXCL10 on receptor activator of nuclear factor kappa-B ligand (RANKL) expression in an animal model of RA and suggested the contribution to osteoclastogenesis. We tested the effects of CXCL10 on the expression of RANKL in RA synoviocytes and T cells, and we investigated which subunit of CXCR3 contributes to RANKL expression by CXCL10.

**Methods:**

Synoviocytes derived from RA patients were kept in culture for 24 hours in the presence or absence of TNF-α. CXCL10 expression was measured by reverse transcriptase polymerase chain reaction (RT-PCR) of cultured synoviocytes. Expression of RANKL was measured by RT-PCR and western blot in cultured synoviocytes with or without CXCL10 and also measured in Jurkat/Hut 78 T cells and CD4+ T cells in the presence of CXCL10 or dexamethasone. CXCL10 induced RANKL expression in Jurkat T cells was tested upon the pertussis toxin (PTX), an inhibitor of Gi subunit of G protein coupled receptor (GPCR). The synthetic siRNA for Gαi_2 _was used to knock down gene expression of respective proteins.

**Results:**

CXCL10 expression in RA synoviocytes was increased by TNF-α. CXCL10 slightly increased RANKL expression in RA synoviocytes, but markedly increased RANKL expression in Jurkat/Hut 78 T cell or CD4+ T cell. CXCL10 augmented the expression of RANKL by 62.6%, and PTX inhibited both basal level of RANKL (from 37.4 ± 16.0 to 18.9 ± 13.0%) and CXCL10-induced RANKL expression in Jurkat T cells (from 100% to 48.6 ± 27.3%). Knock down of Gα_i2 _by siRNA transfection, which suppressed the basal level of RANKL (from 61.8 ± 17.9% to 31.1 ± 15.9%) and CXCL10-induced RANKL expression (from 100% to 53.1 ± 27.1%) in Jurkat T cells, is consistent with PTX, which inhibited RANKL expression.

**Conclusions:**

CXCL10 increased RANKL expression in CD4+ T cells and it was mediated by Gα_i _subunits of CXCR3. These results indicate that CXCL10 may have a potential role in osteoclastogenesis of RA synovial tissue and subsequent joint erosion.

## Introduction

Interferon-gamma (IFN-γ)-inducible protein 10 (CXCL10, also called IP-10) was initially identified as a chemokine that is induced by IFN-γ and secreted by various cell types, such as monocytes, neutrophils, endothelial cells, keratinocytes, fibroblasts, mesenchymal cells, dendritic cells, and astrocytes [1]. CXCL10 is a 10-kDa protein and is functionally categorized as an 'inflammatory' chemokine. Moreover, CXCL10 lacking its ELR motif suppresses neovascularization and functions as an 'angiostatic' chemokine [2]. CXCL10 binds to CXCR3 and regulates immune responses by activating and recruiting leukocytes, such as T cells, eosinophils, monocytes, and natural killer cells [3,4]. Three CXCR3-binding ligands are known, namely CXCL9 (Mig), CXCL10 (IP-10), and CXCL11 (ITAC). Recent reports have shown that the serum or tissue expressions of CXCL10 or both are increased in various autoimmune diseases like rheumatoid arthritis (RA), systemic lupus erythematosus, systemic sclerosis, and multiple sclerosis [5-8], and CXCL10 and CXCR3 may have important roles in leukocytes homing to inflamed tissues and in the perpetuation of inflammation and thus may contribute importantly to tissue damage.

RA is a chronic inflammatory arthritis and is characterized by joint inflammation, synovial hyperplasia, and excessive bone resorption, which are initiated by the recruitment of activated T cells [9]. The regulation of T-cell infiltration into synovium is an important aspect of RA progression. Although it has been reported that many chemokines and proinflammatory cytokines induce the infiltration of inflammatory cells (mainly mononuclear cells and T cells) into the synovium of inflamed joints and thus mediate inflammation [10,11], the etiology of RA remains unknown.

A Th1/Th2 cytokine imbalance with a predominance of Th1 cytokines, including IFN-γ, is suggested to be of pathogenetic importance in RA [12-14]. The Th1 phenotype expresses certain chemokine receptors, including CXCR3 and CCR5 [15,16].

CXCL10 has been detected in sera, synovial fluid, and synovial tissue in patients with RA [5,17]. Furthermore, its concentrations in RA synovial fluid have been reported to be higher than in osteoarthritis (OA) synovial fluid and higher than serum concentrations in patients with RA [5]. CXCL10 is expressed mainly by infiltrating macrophage-like cells and fibroblast-like synoviocytes in RA synovium [5,18].

In our previous animal experiment, receptor activator of nuclear factor-kappa B ligand (RANKL) induced CXCL10 expression on osteoclast precursors, and, reciprocally, CXCL10 upregulated RANKL expression in CD4^+ ^T cells [18]. To examine the potential role of CXCL10 in real osteoclastogenesis, osteoclast precursors were cocultured with CD4^+ ^T cells in the presence of CXCL10, and it was found that CXCL10 induced TRAP (tartrate-resistant acid phosphatase)-positive osteoclast differentiation in a dose-dependent manner. Furthermore, this differentiation induced by CXCL10 was suppressed by osteoprotegerin (OPG) (a soluble RANKL antagonist) and by neutralizing anti-CXCL10 antibody [18]. The treatment of collagen-induced arthritis mice with neutralizing anti-CXCL10 antibody not only suppressed arthritis progression but also attenuated histological bone loss [18]. Therefore, the purpose of this study was to investigate the effects of CXCL10 on the expression of RANKL in human RA synoviocytes and CD4^+ ^T cells and to find which subunit of CXCR3 contributes to RANKL expression by CXCL10.

## Materials and methods

### Synovial fluid and sera

Synovial fluid and sera were collected from 18 patients who had RA and who fulfilled the 1987 American College of Rheumatology (ACR) criteria for RA and 11 patients who had OA and who fulfilled ACR criteria for knee OA. This study was approved by the institutional review board of the Seoul National University College of Medicine, and informed consent was obtained from all of the sample donors in this study.

### Reagent

Human CXCL10 was purchased from PeproTech (Rocky Hill, NJ, USA) and TNF-α and interleukin-1-beta were purchased from R&D Systems, Inc. (Minneapolis, MN, USA). Pertussis toxin (PTX) was purchased from Calbiochem (now part of EMD Biosciences, Inc., San Diego, CA, USA).

### Cell culture

Synoviocytes were obtained from three patients who had RA and who underwent total knee replacement arthroplasty. The synoviocytes were cultured for 24 hours in the presence or absence of TNF-α (20 ng/mL). We used two kinds of T-cell lines: Jurkat T cells and Hut 78 T cells. These cells were cultured in the presence of CXCL10 (10 or 100 ng/mL) or dexamethasone (10^-7 ^M). CD4^+ ^T cells derived from healthy donors were isolated from peripheral blood mononuclear cells by magnetic-activated cell sorting (MACS) and cultured for 6 hours in the presence of CXCL10 (100 ng/mL) or dexamethasone (10^-7 ^M).

### Measurement of CXCL10

CXCL10 concentrations of synovial fluid and sera were measured by a commercial enzyme-linked immunosorbent assay (ELISA) kit (R&D Systems, Inc.).

### Expression of CXCL10, RANK, and RANKL

CXCL10 expression in RA synoviocytes was measured by reverse transcriptase-polymerase chain reaction (RT-PCR) using primers (forward: TGACTCTAAGTGGCATTCAAGG; reverse: GATTCAGACATCTCTTCTCACCC) and by Western blotting using anti-CXCL10 antibody (R&D Systems, Inc.). RANKL expressions in synoviocytes, Jurkat T cells, Hut 78 T cells, and CD4^+ ^T cells were measured by RT-PCR using primers (forward: GCCAGTGGGAGATGTTAG; reverse: TTAGCTGCAAGTTTTCCC), by real-time polymerase chain reaction with Taqman probe, and by Western blotting using anti-RANKL antibody purchased from R&D Systems, Inc. RANK expression in CD14^+ ^monocytes from healthy donors in the presence of MCSF with or without CXCL10 was measured by RT-PCR using primers (forward: TTAAGCCAGTGCTTCACGGG; reverse: ACGTAGACCACGATGATGTCGC). Levels of OPG in cultured RA synoviocytes were measured by using a commercial ELISA kit (R&D Systems, Inc.).

### RNA interference

The synthetic small interfering RNA (siRNA) for Gα_i2 _and control (Santa Cruz Biotechnology, Inc., Santa Cruz, CA, USA) were used to knock down gene expression of respective proteins. The siRNA transfection was performed by electroporation using Gene Pulser II (Bio-Rad Laboratories, Inc., Hercules, CA, USA) at 250 V/950 F.

### Immunoblot analysis

Cells were harvested and lysed in a lysis buffer (Cell Signaling Technology, Inc., Danvers, MA, USA) by incubating the suspension on ice for 20 minutes. The protein concentration of the lysate was measured using the bicinchoninic acid method. Fifty micrograms of the lysate protein was boiled in a Lammli buffer, separated on a 10% SDS polyacrylamide gel, and then transferred to a nitrocellulose membrane. The blot was blocked with 5% non-fat milk for 1 hour and then incubated in a cold room overnight with a specific antibody. The primary antibodies used were as follows: antibody against RANKL; antibody against Gα_i2 _from Santa Cruz Biotechnology, Inc.; antibody against phosphorylated CREB (cAMP-response element binding) (Ser133) from Cell Signaling Technology, Inc.; and actin antibody from Sigma-Aldrich (St. Louis, MO, USA). The nitrocellulose membrane was subsequently washed and incubated with a peroxidase-labeled rabbit anti-goat IgG antibody for 2 hours at room temperature and then incubated with an enhanced chemiluminescence substrate mixture (Pierce, Rockford, IL, USA). The blot was then exposed on x-ray film (AGFA Curix RPI; Agfa HealthCare NV, Mortsel, Belgium) to obtain an image. Densities of visualized bands were quantified using an image analyzer (Model Multi Gauge V2.3; Fujifilm, Tokyo, Japan).

## Results

CXCL10 concentrations were increased in patients with RA and CXCL10 expressions in RA synoviocytes were increased by TNF-α

**Figure 1:**
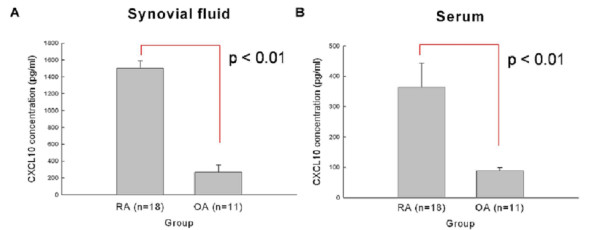
**Comparison of CXCL10 concentrations between patients with rheumatoid arthritis (RA) and those with osteoarthritis (OA)**. CXCL10 concentrations of synovial fluid **(a) **and sera **(b) **were measured by enzyme-linked immunosorbent assay. CXCL10 concentrations were significantly increased in synovial fluid and sera of RA in comparison with those of OA. Data were expressed as mean ± standard error. CXCL10, interferon-gamma-inducible protein 10.

**Figure 2:**
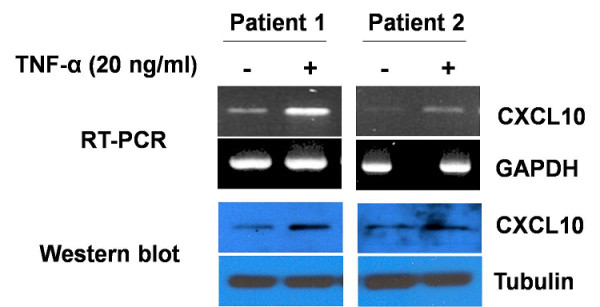
**CXCL10 expressions in rheumatoid arthritis (RA) synoviocytes (Western blot and reverse transcriptase-polymerase chain reaction)**. Human RA synoviocytes were cultured in the presence or absence of tumor necrosis factor-alpha (TNF-α) for 24 hours. Without TNF-α stimulation, low expressions of CXCL10 were observed in RA synoviocytes with high individual variation. However, with TNF-α stimulation, CXCL10 expression in RA synoviocytes was significantly increased. CXCL10, interferon-gamma-inducible protein 10; GAPDH, glyceraldehyde-3-phosphate dehydrogenase; RT-PCR, reverse transcriptase-polymerase chain reaction.

We compared CXCL10 concentration of synovial fluids and sera in 18 patients with RA and 11 patients with OA. Demographic data of both sets of patients at the time of sampling are presented in Table 1. As shown in Figure 1, CXCL10 concentrations were significantly increased in RA synovial fluid (mean ± standard error: 1,502.0 ± 87.1 pg/mL versus 267.3 ± 87.0 pg/mL; *P *< 0.01) and sera (363.9 ± 78.9 pg/mL versus 87.7 ± 10.8 pg/mL; *P *< 0.01) than in those of OA. Furthermore, the concentration of CXCL10 in inflamed synovial fluid is much higher than in sera of patients with RA (1,502.0 ± 87.1 pg/mL versus 363.9 ± 78.9 pg/mL; *P *< 0.05). Although the baseline expression level of CXCL10 varied in individual patients, CXCL10 expression in RA synoviocytes was increased by TNF-α (Figure 2).

**Table 1:** Demographic data of patients with osteoarthritis and those with rheumatoid arthritis

	Osteoarthritis (*n *= 11)	Rheumatoid arthritis (*n *= 18)
Age in years, mean ± SD	67.20 ± 7.35	57.17 ± 9.51
Females/Males	10:1	17:1
Duration of disease in months, mean ± SD	79.64 ± 62.66	114.85 ± 49.54
Treatment, number (percentage)		
Prednisolone		15 (83%)
DMARDs		18 (100%)
Methotrexate		16 (89%)
Sulfasalazine		2 (11%)
Hydroxychloroguine		7 (39%)
Leflunomide		5 (28%)
Azathioprine		3 (17%)

DMARD, disease-modifying antirheumatic drug; SD, standard deviation.

### CXCL10 induced RANKL in CD4^+ ^T cells and synoviocytes

**Figure 3:**
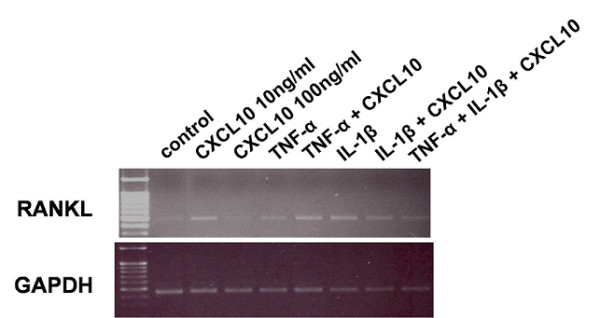
**Induction of receptor activator of nuclear factor kappa-B ligand (RANKL) by CXCL10 in rheumatoid arthritis (RA) synoviocytes (reverse transcriptase-polymerase chain reaction)**. CXCL10 (10 ng/mL) induced RANKL in RA synoviocytes. Tumor necrosis factor-alpha (TNF-α) (10 ng/mL) alone weakly induced RANKL expression in this sample. However, when CXCL10 was added to TNF-α, RANKL expression increased. Interleukin-1-beta (IL-1β) (10 ng/mL) alone induced RANKL gene expression, and band intensity did not change when CXCL10 was added. CXCL10, interferon-gamma-inducible protein 10; GAPDH, glyceraldehyde-3-phosphate dehydrogenase.

**Figure 4:**
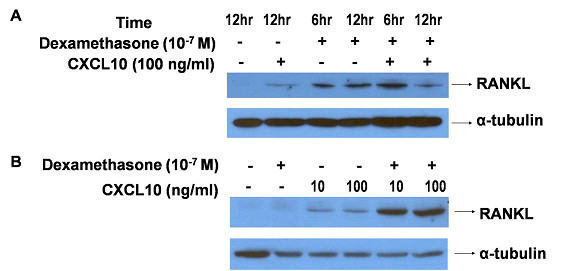
**Induction of receptor activator of nuclear factor kappa-B ligand (RANKL) by CXCL10 in T-cell lines (Western blot)**. CXCL10 increased RANKL protein expression in Jurkat T cells **(a) **and Hut 78 T cells **(b)**. Dexamethasone was used as a positive control in this experiment, and dexamethasone-induced RANKL expression was potentiated by the addition of CXCL10. CXCL10, interferon-gamma-inducible protein 10.

**Figure 5:**
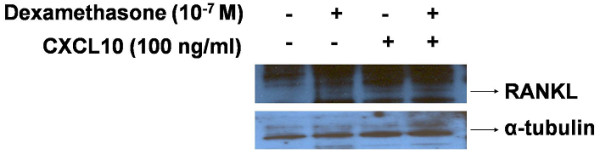
**Induction of receptor activator of nuclear factor kappa-B ligand (RANKL) by CXCL10 in CD4^+ ^T cells (Western blot)**. Human CD4^+ ^cells from healthy donors were used in this experiment, and CXCL10 increased RANKL protein expression in human primary CD4^+ ^T cells. CXCL10, interferon-gamma-inducible protein 10.

We found that CXCL10 could induce RANKL in RA synoviocytes but that RANKL expression induced by CXCL10 was relatively weak (Figure 3) and showed high individual variation (Additional file 1). We also checked the effect of CXCL10 on RANKL expression in T cells because activated T cells could express RANKL and contribute to osteoclastogenesis in RA. As shown in Figure 4, CXCL10 increased RANKL expression in Jurkat T cells (A) and Hut 78 T cells (B). Dexamethasone-induced RANKL expression was potentiated by the addition of CXCL10. We checked RANKL expression in primary CD4^+ ^T cells from healthy donors in the presence or absence of CXCL10 and found that CXCL10 independently could induce RANKL in human CD4^+ ^T cells (Figure 5). We searched the effect of CXCL10 on RANK expression on CD14^+ ^monocytes and OPG level in RA synoviocytes (Additional file 2). Finally, there was no significant effect on RANK expression in monocytes after CXCL10 stimulation. CXCL10 stimulation did not significantly induce or reduce OPG production in cultured synoviocytes or Jurkat T cells.

### Gα_i _subunits were involved in CXCL10-induced RANKL expression in jurkat T cells

**Figure 6:**
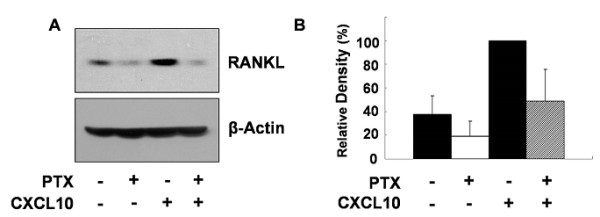
**Effect of pertussis toxin (PTX) on CXCL10-induced expression of receptor activator of nuclear factor kappa-B ligand (RANKL)**. Expression was assessed by immunoblot assay **(a) **and densitometry **(b)**. Jurkat T cells were pretreated with 200 ng/mL PTX for 18 hours and then treated with 10 ng/mL CXCL10 for 6 hours. RANKL expression was assessed by immunoblot analysis using a specific antibody against RANKL. The immonoblot assays shown are representative of at least four independent experiments, and the histograms show average and standard deviations of representative RANKL expression. CXCL10, interferon-gamma-inducible protein 10.

**Figure 7:**
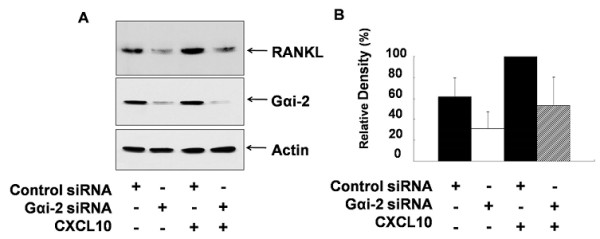
**Effect of Gα_i2 _siRNA (small interfering RNA) on CXCL10-induced expression of receptor activator of nuclear factor kappa-B ligand (RANKL)**. Jurkat T cells were transfected with Gα_i2 _and control siRNA by electroporation. After 48 hours, the cells were treated with 10 ng/mL CXCL10 for 6 hours, and then RANKL expression was assessed by immunoblot analysis using a specific antibody against RANKL **(a)**. The bolts shown are representative of at least four independent experiments, and the histograms show average and standard deviations of representative RANKL expression **(b)**. CXCL10, interferon-gamma-inducible protein 10.

To investigate whether Gα_i _subunits are involved in CXCL10-induced RANKL expression, we used PTX, a bacterial toxin that inhibits Gα_i _activation by ADP-ribosylating Gα_i _subunits, and the inhibition of Gα_i _by PTX was confirmed by measuring CREB phosphorylation that was increased following Gα_i _inhibition. Figure 6 shows that CXCL10 augmented the expression of RANKL more than twofold and that PTX inhibited both basal level of RANKL (from 37.4% ± 16.0% to 18.9% ± 13.0%) and CXCL10-induced RANKL expression in Jurkat T cells (from 100% to 48.6% ± 27.3%). Next, to prove the involvement of Gα_i _in regulating CXCL10-induced RANKL expression, we analyzed the effect of Gα_i2 _siRNA on CXCL10-induced RANKL expression. Knockdown of Gα_i2 _by siRNA transfection, which suppressed the basal level of RANKL (from 61.8% ± 17.9% to 31.1% ± 15.9%) and CXCL10-induced RANKL expression (from 100% to 53.1 ± 27.1%) in Jurkat T cells, is consistent with PTX, which inhibited RANKL expression (Figure 7). These results indicate that Gα_i _subunits are involved in CXCL10-induced RANKL expression in Jurkat T cells.

## Discussion

The regulation of osteoclasts is vital for maintaining balance in bone remodeling. Bone resorbing osteoclasts are derived from hemopoeitic cells of the monocyte/macrophage lineage and differentiate into multinucleated cells through multiple processes [19]. Osteoclast formation and activity are regulated by local factors and by stromal and osteoblast cells in the bone environment [20]. Several chemokines promote bone resorption by inducing osteoclast formation and survival and by directly inducing the migration and adhesion of leukocytes [21,22]. For example, the expressions of macrophage inflammatory protein-1α (MIP-1α, CCL3) and MIP-1β (CCL4) in multiple myeloma cells were found to enhance osteolytic lesions by enhancing osteoclast formation and bone resorption, and, more recently, CCR2 was shown to increase expression of RANK and enhance RANK signaling in ovariectomized mice [23,24]. Of the several osteoclastogenic factors, RANKL, which is expressed on stromal and osteoblast cells, is known to play an essential role in osteoclast differentiation and function. Furthermore, the regulation of RANKL expression is known to be important for preventing bone disorders caused by increased osteoclast formation.

In our experiments, CXCL10 increased RANKL expression in RA synoviocytes and directly increased RANKL expression in Jurkat/Hut 78 T cells or human CD4^+ ^T cells. These results are compatible with our previous animal experiments showing that CXCL10 upregulated RANKL expression in mouse T cells and induced TRAP-positive osteoclast differentiation [18]. In both human and mouse systems, CXCL10 can induce RANKL expression, mainly in CD4^+ ^T cells, and then may contribute to osteoclastogenesis.

CXCL10-induced RANKL expression in human T cells seems to be mediated by Gα_i _subunits of CXCR3, which is a well-known receptor for CXCL9, CXCL10, and CXCL11. CXCR3 is a G protein-coupled, seven-transmembrane receptor and has heterotrimeric G proteins consisting of α, β, and γ subunits. The G protein resides attached to the intracellular face of the plasma membrane in an inactive form consisting of the Gα subunit bound to GDP, a structure that is stabilized by interaction with the βγ dimer. Upon interaction with the receptor, Gα protein becomes activated, causing GDP exchange for GTP. The GTP-binding proteins are classified by the signaling events they instigate, of which there are four major families: Gα_i/o_, Gα_q/11_, Gα_12/13_, and Gα_s _[25,26]. T lymphocytes usually express Gα_i_, which plays a major role in chemotaxis, proliferation, and differentiation of various cells [27,28]. In our experiment, depletion of Gα_i _subunit by siRNA or PTX suppressed the RANKL basal level and CXCL10-induced RANKL expression in Jurkat T cells. These results suggest that the Gα_i _subunit of CXCR3 in T lymphocytes mediates CXCL10-induced RANKL expression and may contribute to osteoclastogenesis.

Our data showed that CXCL10 levels in sera and synovial fluid in patients with RA were significantly higher than those of patients with OA. CXCL10 expression in RA synoviocytes is upregulated by TNF-α, which is a major pathologic cytokine in RA. The increased amount of CXCL10 and TNF-α may recruit osteoclast precursor cells, induce RANKL, and then induce osteoclastogenesis. This kind of auto- or paracrine amplification loop may contribute to chronic bone destructive damage in inflamed RA joints.

## Conclusions

CXCL10 increased RANKL expression in CD4^+ ^T cells and was mediated by Gα_i _subunits of CXCR3. In addition to having a role in the recruitment of proinflammatory cells, CXCL10 may have a potential role in osteoclastogenesis of RA synovial tissue and subsequent joint erosion.

## Abbreviations

ACR: American College of Rheumatology; CREB: cAMP-response element binding; CXCL10: interferon-gamma-inducible protein 10 (IP-10); ELISA: enzyme-linked immunosorbent assay; IFN-γ: interferon-gamma; MACS: magnetic-activated cell sorting; MIP-1: macrophage inflammatory protein 1; OA: osteoarthritis; OPG: osteoprotegerin; PTX: pertussis toxin; RA: rheumatoid arthritis; RANK: receptor activator of nuclear factor-kappa B; RANKL: receptor activator of nuclear factor-kappa B ligand; RT-PCR: reverse transcriptase-polymerase chain reaction; siRNA: small interfering RNA; TNF: tumor necrosis factor; TRAP: tartrate-resistant acid phosphatase.

## Competing interests

The authors declare that they have no competing interests.

## Authors' contributions

YWS is principal investigator and EYL is chief investigator for this study. YJL and EBL contributed to the conception of the study, the interpretation of the data, and the writing of the Discussion section. MRS and Y-SJ contributed to RNA interference and immunoblot analysis. JYK and YJH contributed to cell isolation, culture, RT-PCR, and immunoblot analysis. All authors read and approved the final manuscript.

## Supplementary Material

Additional file 1 **Receptor activator of nuclear factor kappa-B ligand (RANKL) induction by CXCL10 in rheumatoid arthritis (RA) synoviocytes and Jurkat T cell (real-time PCR)**. RANKL induction by CXCL10 in RA synoviocytes (*n *= 3) showed high individual variation but there was an increasing tendency of RANKL expression by CXCL10 stimulation. In Jurkat T cell, CXCL10 highly increased RANKL expression. Click here for file

Additional file 2 **Effect of CXCL10 on receptor activator of nuclear factor kappa-B (RANK) expression in CD14^+ ^mococytes (reverse transcriptase-polymerase chain reaction)**. CD14^+ ^mococytes derived from healthy donor were isolated by MACS microbeads and cultured in the presence of MCSF (25 ng/mL) with or without CXCL10 (10 ng/mL or 100 ng/mL). After 24 or 48 hour, the cells were collected and then RANK expression was assessed by RT-PCR (A). Effect of CXCL10 on osteoprotegerin (OPG) production in rheumatoid arthritis (RA) synoviocytes (enzyme-linked immunosorbent assay). RA synoviocytes (*n *= 3) were cultured in the presence or absence of CXCL10 (10 ng/mL or 100 ng/mL) for 24 or 48 hours and then, culture media were collected for measurement of OPG. Click here for file

## References

[B1] Luster AD Ravetch JV Biochemical characterization of a gamma interferon- inducible cytokine (IP-10) J Exp Med 1987 166 1084 1097 10.1084/jem.166.4.1084 2443596 PMC2188708

[B2] Strieter RM Polverini PJ Kunkel SL Arenberg DA Burdick MD Kasper J Dzuiba J Damme JV Walz A Marriott D Chan SY Roczniak S Shanafelt AB The functional role of the ELR motif in CXC chemokine-mediated angiogenesis J Biol Chem 1995 270 27348 27357 10.1074/jbc.270.45.27348 7592998

[B3] Taub DD Lioyd AR Conlon K Wang JM Ortaldo JR Harada A Matsushima K Kelvin DJ Oppenheim JJ Recombinant human interferon-inducible protein 10 is a chemoattractant for human monocytes and T lymphocytes and promotes T cell adhesion to endothelial cells J Exp Med 1993 177 1809 1814 10.1084/jem.177.6.1809 8496693 PMC2191047

[B4] Jinquan T Jing C Jacobi HH Reimert CM Millner A Quan S Madsen HO Ryder LP Svejgaard A Malling HJ Skov PS Poulsen LK CXCR3 expression and activation of eosinophils: role of IFN-gamma-inducible protein-10 and monokine induced by IFN-gamma J Immunol 2000 165 1548 1556 10903763 10.4049/jimmunol.165.3.1548

[B5] Hanaoka R Kasama T Muramatsu M Yajima N Shiozawa F Miwa Y Negishi M Ide H Miyaoka H Uchida H Adachi M A novel mechanism for the regulation of IFN-γ-inducible protein-10 expression in rheumatoid arthritis Arthritis Res Ther 2003 5 R74 81 10.1186/ar616 12718750 PMC165028

[B6] Narumi S Takeuchi T Kobayashi Y Konish K Serum levels of ifn-inducible PROTEIN-10 relating to the activity of systemic lupus erythematosus Cytokine 2000 12 1561 1565 10.1006/cyto.2000.0757 11023674

[B7] Fujii H Shimada Y Hasegawa M Takehara K Kamatain N Serum levels of a Th1 chemoattractant IP-10 and Th2 chemoattractants, TARC and MDC, are elevated in patients with systemic sclerosis J Dermatol Sci 2004 35 43 51 10.1016/j.jdermsci.2004.03.001 15194146 15194146

[B8] Sørensen TL Tani M Jesnsen J Pierce V Lucchinetti C Folcik VA Qin S Rottman J Sellebjerg F Strieter RM Frederiksen JL Ransohoff RM Expression of specific chemokines and chemokine receptors in the central nervous system of multiple sclerosis patients J Clin Invest 1999 103 807 815 10.1172/JCI5150 10079101 PMC408141

[B9] O'Gradaigh D Compston JE T-cell involvement in osteoclast biology: implications for rheumatoid bone erosion Rheumatology (Oxford) 2004 43 122 130 12867576 10.1093/rheumatology/keg447

[B10] Nanki T Hayashida K El-Gabalawy HS Suson S Shi K Girschick HJ Yavuz S Lipsky PE Stromal cell-derived factor-1-CXC chemokine receptor 4 interactions play a central role in CD4+ T cell accumulation in rheumatoid arthritis synovium J Immunol 2000 165 6590 6598 11086103 10.4049/jimmunol.165.11.6590

[B11] Ji H Pettit A Ohmura K Ortiz-Lopez A Duchatelle V Degott C Gravallese E Mathis D Benoist C Critical roles for interleukin 1 and tumor necrosis factor alpha in antibody-induced arthritis J Exp Med 2002 196 77 85 10.1084/jem.20020439 12093872 PMC2194010

[B12] Yin Z Siegert S Neure L Grolms M Liu L Eggens U Radbruch A Braun J Sieper J The elevated ratio of interferon gamma-/interleukin-4-positive T cells found in synovial fluid and synovial membrane of rheumatoid arthritis patients can be changed by interleukin-4 but not by interleukin-10 or transforming growth factor beta Rheumatology (Oxford) 1999 38 1058 1067 10.1093/rheumatology/38.11.1058 10556256

[B13] Canete JD Martinez SE Farres J Sanmarti R Blay M Gomez A Salvador G Munoz-Gomez J Differential Th1/Th2 cytokine patterns in chronic arthritis: interferon gamma is highly expressed in synovium of rheumatoid arthritis compared with seronegative spondyloarthropathies Ann Rheum Dis 2000 59 263 268 10.1136/ard.59.4.263 10733472 PMC1753106

[B14] Kotake S Nanke Y Mogi M Kawamoto M Furuya T Yago T Koashigawa T Togari A Kamatani N IFN-gamma-producing human T cells directly induce osteoclastogenesis from human monocytes via the expression of RANKL Eur J Immunol 2005 35 3353 3363 10.1002/eji.200526141 16220542

[B15] Sallusto F Lenig D Mackay CR Lanzavecchia A Flexible programs of chemokine receptor expression on human polarized T helper 1 and 2 lymphocytes J Exp Med 1998 187 875 883 10.1084/jem.187.6.875 9500790 PMC2212187

[B16] Rossi D Zlotnik A The biology of chemokines and their receptors Annu Rev Immunol 2000 18 217 242 10.1146/annurev.immunol.18.1.217 10837058

[B17] Patel DD Zachariah JP Whichard LP CXCR3 and CCR5 ligands in rheumatoid arthritis synovium Clin Immunol 2001 98 39 45 10.1006/clim.2000.4957 11141325

[B18] Kwak HB Ha H Kim HN Lee JH Kim HS Lee S Kim HM Kim JY Kim HH Song YW Lee ZH Reciprocal cross-talk between RANKL and interferon-gamma-inducible protein 10 is responsible for bone-erosive experimental arthritis Arthritis Rheum 2008 58 1332 1342 10.1002/art.23372 18438854

[B19] Teitelbaum SL Ross FP Genetic regulation of osteoclast development and function Nat Rev Genet 2003 4 638 649 10.1038/nrg1122 12897775

[B20] Krane SM Identifying genes that regulate bone remodeling as potential therapeutic targets J Exp Med 2005 201 841 843 10.1084/jem.20050354 15781576 PMC2213103

[B21] Wright LM Maloney W Yu X Kindle L Collin-Osdoby P Osdoby P Stromal cell-derived factor-1 binding to its chemokine receptor CXCR4 on precursor cells promotes the chemotactic recruitment, development and survival of human osteoclasts Bone 2005 36 840 853 10.1016/j.bone.2005.01.021 15794931

[B22] Kwak HB Lee SW Jin HM Ha H Lee SH Takeshita S Tanaka S Kim HM Kim HH Lee ZH Monokine induced by interferon-gamma is induced by receptor activator of nuclear factor kappa B ligand and is involved in osteoclast adhesion and migration Blood 2005 105 2963 2969 10.1182/blood-2004-07-2534 15585657

[B23] Abe M Hiura K Wilde J Moriyama K Hashimoto T Ozaki S Wakatsuki S Kosaka M Kido S Inoue D Matsumoto T Role for macrophage inflammatory protein (MIP)-1alpha and MIP-1beta in the development of osteolytic lesions in multiple myeloma Blood 2002 100 2195 2202 12200385

[B24] Binder NB Niederreiter B Hoffmann O Stange R Pap T Stulnig TM Mack M Erben RG Smolen JS Redlich K Estrogen-dependent and C-C chemokine receptor-2-dependent pathways determine osteoclast behavior in osteoporosis Nat Med 2009 15 417 424 10.1038/nm.1945 19330010

[B25] Wilkie TM Gilbert DJ Olsen AS Chen X Amatruda TT Korenberg JR Trask BJ de Jong P Reed RR Simon MI Jenkins NA Copeland NG Evolution of the mammalian G protein alpha subunit multigene family Nat Genet 1992 1 85 91 10.1038/ng0592-85 1302014

[B26] Fields TA Casey PJ Signalling functions and biochemical properties of pertussis toxin-resistant G-proteins Biochem J 1997 321 561 571 9032437 10.1042/bj3210561 PMC1218106

[B27] Kaslow HR Burns DL Pertussis toxin and target eukaryotic cells: binding, entry, and activation FASEB J 1992 6 2684 2690 1612292 10.1096/fasebj.6.9.1612292

[B28] Kehrl JH Heterotrimeric G protein signaling: roles in immune function and fine-tuning by RGS proteins Immunity 1998 8 1 10 10.1016/S1074-7613(00)80453-7 9462506

