# Supplementary figures and images for: Potential role and mechanism of IFN-gamma inducible protein-10 on receptor activator of nuclear factor kappa-B ligand (RANKL) expression in rheumatoid arthritis
Source: Arthritis Res Ther. 2011 Jun 27;13(3):R104. doi: 10.1186/ar3385 (PMC3218919; doi:10.1186/ar3385)

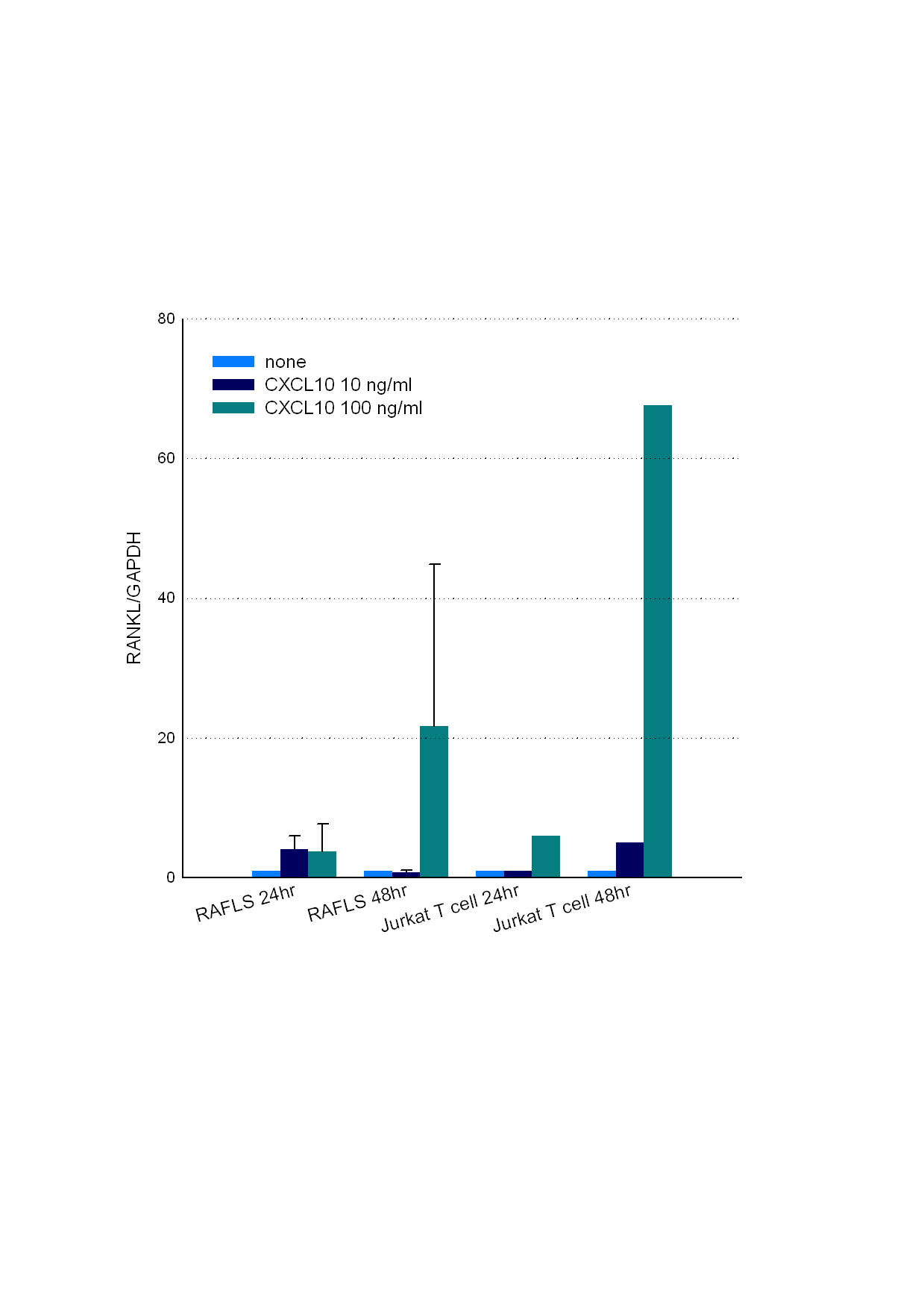

Supplement: Additional file 1 — Receptor activator of nuclear factor kappa-B ligand (RANKL) induction by CXCL10 in rheumatoid arthritis (RA) synoviocytes and Jurkat T cell (real-time PCR). RANKL induction by CXCL10 in RA synoviocytes (n = 3) showed high individual variation but there was an increasing tendency of RANKL expression by CXCL10 stimulation. In Jurkat T cell, CXCL10 highly increased RANKL expression. [file ar3385-S1.JPEG]

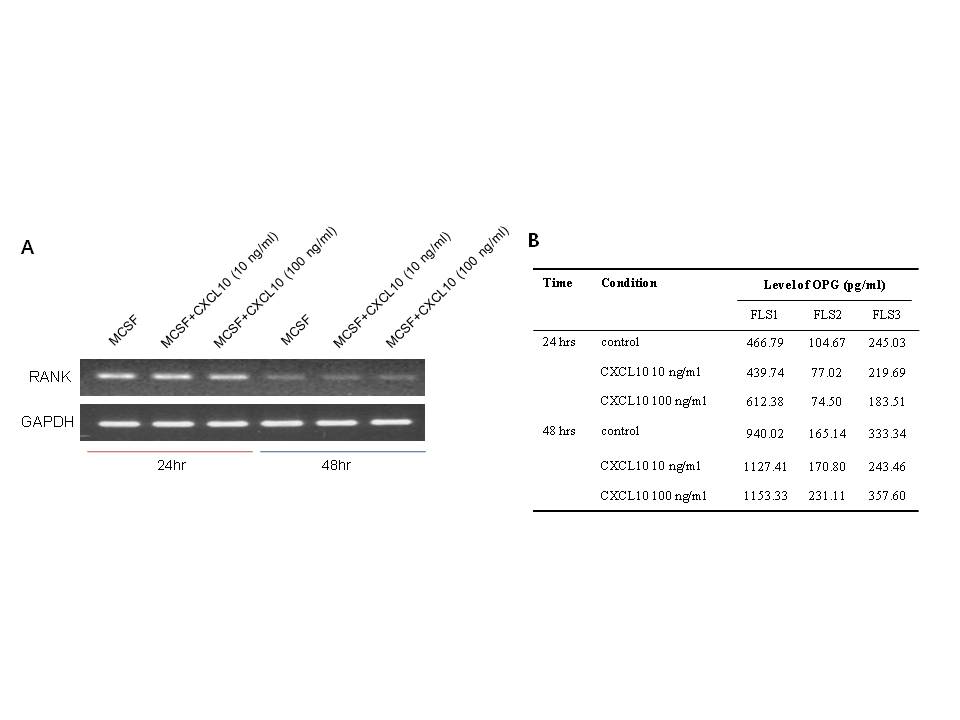

Supplement: Additional file 2 — Effect of CXCL10 on receptor activator of nuclear factor kappa-B (RANK) expression in CD14+ mococytes (reverse transcriptase-polymerase chain reaction). CD14+ mococytes derived from healthy donor were isolated by MACS microbeads and cultured in the presence of MCSF (25 ng/mL) with or without CXCL10 (10 ng/mL or 100 ng/mL). After 24 or 48 hour, the cells were collected and then RANK expression was assessed by RT-PCR (A). Effect of CXCL10 on osteoprotegerin (OPG) production in rheumatoid arthritis (RA) synoviocytes (enzyme-linked immunosorbent assay). RA synoviocytes (n = 3) were cultured in the presence or absence of CXCL10 (10 ng/mL or 100 ng/mL) for 24 or 48 hours and then, culture media were collected for measurement of OPG. [file ar3385-S2.JPEG]
